# *Shewanella algae* and *Morganella morganii* Coinfection in Cobra-Bite Wounds: A Genomic Analysis

**DOI:** 10.3390/life11040329

**Published:** 2021-04-10

**Authors:** Wei-Hsuan Huang, Chin-Chuan Kao, Yan-Chiao Mao, Chih-Sheng Lai, Kuo-Lung Lai, Chung-Hsu Lai, Chien-Hao Tseng, Yao-Ting Huang, Po-Yu Liu

**Affiliations:** 1 Division of Infectious Diseases, Department of Internal Medicine, Taichung Veterans General Hospital, Taichung 40705, Taiwan; hpluffy@vghtc.gov.tw (W.-H.H.); tedi3tedi3@vghtc.gov.tw (C.-H.T.); 2 Division of Infectious Disease, Department of Internal Medicine, Tungs’ Taichung Metroharbor Hospital, Taichung 43503, Taiwan; t6925@ms.sltung.com.tw; 3 Division of Clinical Toxicology, Department of Emergency Medicine, Taichung Veterans General Hospital, Taichung 40705, Taiwan; nilnil.nilnil@vghtc.gov.tw; 4 School of Medicine, National Defense Medical Center, Taipei 11490, Taiwan; 5 Division of Plastic and Reconstructive Surgery, Department of Surgery, Taichung Veterans General Hospital, Taichung 40705, Taiwan; laics@vghtc.gov.tw; 6 Division of Allergy, Immunology and Rheumatology, Department of Internal Medicine, Taichung Veterans General Hospital, Taichung 40705, Taiwan; kllaichiayi@vghtc.gov.tw; 7 Division of Infectious Diseases, Department of Internal Medicine, E-Da Hospital, Kaohsiung 840, Taiwan; ed101746@edah.org.tw; 8 School of Medicine, College of Medicine, I-Shou University, Kaohsiung 804, Taiwan; 9 Department of Computer Science and Information Engineering, National Chung Cheng University, Chia-Yi 62102, Taiwan; 10 Ph.D. Program in Translational Medicine, National Chung Hsing University, Taichung 402, Taiwan; 11 Rong Hsing Research Center for Translational Medicine, National Chung Hsing University, Taichung 402, Taiwan

**Keywords:** *Naja atra*, *Shewanella algae*, *Morganella morganii*, coinfection, whole genome sequencing

## Abstract

*Naja atra* bites cause severe soft tissue injury and are prone to wound infections. The pathogens of *Naja atra* bite-wound infections are highly variable in different geographical regions. Here, we report the first coinfection with *Shewanella algae* and *Morganella morganii* from a *Naja atra* bite wound with resistome analysis using whole genome sequencing.

## 1. Introduction

Snakebite is a major neglected tropical disease with significant mortality and morbidity. It is estimated that 4.5–5.4 million people get bitten by snakes annually. Among these patients, 1.8–2.7 million develop clinical illness, and 81,000–138,000 die from complications [1,2]. Snakebite is also a medical problem in Taiwan where the climate ranges from humid subtropical to tropical monsoon and where human activities and animal habitats often overlap due to the high population density, with 672.6 persons per square kilometer [3].

Although *Naja atra* ranks third among the six most common venomous snake bites (*Trimeresurus stejnegeri*, *Bungarus multicinctus*, *N. atra*, *Protobothrops mucrosquamatus*, *Deinagkistrodon acutus*, *Daboia siamensis*) in Taiwan [4], it causes more severe bacterial infection than other kinds of snakebites. The snake’s oral cavity harbors more pathogenic bacteria associated with wound infection. The most commonly reported pathogenic bacteria consisted of various Gram-negative and Gram-positive bacterium, and there is considerable geographical variability in the cobra-bite wound infection pathogens [5]. Although coinfection is not uncommon in bite-wound infection from other animals, there have been few reports of coinfection of snake bite wounds.

Our current understanding of the epidemiology and clinical manifestations of *Shewanella* is derived from a limited number of case reports [6]. Published data and clinical information about *Shewanella* infections related to snakebites also remain scarce. To the best of our knowledge, this is the first case report of coinfection with *Shewanella algae* and *Morganella morganii* in a wound caused by *N. atra* bite with resistome analysis using whole genome sequencing.

## 2. Case Presentation

A 61-year-old man with hypertension and hyperuricemia was transferred to the emergency department 20 minutes after being bitten by a cobra (*N. atra*) in his house. The snake was captured and identified by specialist, Dr. Yan-Chiao Mao. The bite had resulted in two puncture wounds on the lateral dorsal surface of his left foot. Local swelling, heat, and pain over his left foot extending to the medial thigh developed.

His vital signs revealed a heart rate of 119 beats/min, blood pressure of 117/78 mmHg, respiratory rate of 20 breaths/min, and temperature of 37 °C. A pain score of eight points was determined on a numerical rating scale. Seven hours after sustaining the snakebite, fever developed, reaching a temperature of 38.6 °C. Vomiting and diarrhea more than ten times/day developed 16 hours after being bitten. The patient was able to move his left leg, but the range of motion was limited. Physical examination revealed an extremely swollen left leg with edematous, purple-colored skin, and some bullae on the soft tissue over the left dorsal foot and left ankle joint.

Blood tests indicated that his peripheral leukocyte count was 7.3 × 10^9^/L (3.9–10.6 × 10^9^/L) with 72.0% segmented neutrophils, and hematocrit of 39.4%. His platelet count was 204 × 10^9^/L (150–400 × 10^9^/L). C-reactive protein was 32.98 mg/L (<0.3 mg/L). Additionally, aspartate aminotransferase was 72 U/L (normal: < 37 U/L) and serum creatinine was 1.46 mg/dl (normal: 0.4–1.4 mg/dL). Computed tomography (CT) of the left leg performed with contrast material showed reticular pattern infiltration in the subcutaneous tissue over the left dorsal foot, ankle, and the anterolateral aspect of the left lower leg. There was not subcutaneous air, intramuscular abscess, nor destruction of bones.

*S. algae* and *M. morganii* were isolated from a conventional culture of aspirated bulla fluid. Intravenous piperacillin–tazobactam was administered, and intravenous antivenom was injected at 20 min, 3 h, 13 h, 37 h, and 44 h after snake bite, totaling eight vials. The local symptoms progressed rapidly over the next 2 days. He underwent surgical fasciotomy and extensive debridement of soft tissue along the dorsalis pedis and lateral side of the tibialis. Soft tissue specimens were obtained and sent for conventional culture. *S. algae* was designated as VGH 117, and *M. morganii* was designated as VGH 116. The minimum inhibitory concentrations (MICs) of *M. morganii* VGH116 and *S. algae* VGH117 are summarized in Table 1.

The patient subsequently underwent debridement four times after the first operation. The wound condition improved slowly after debridement, and a skin graft was performed on day 22 after sustaining the snakebite. The patient was discharged from the hospital on day 37 post-bite.

## 3. Materials and Methods

### 3.1. Genome Sequencing and Assembly

The bacterial genomic DNA was extracted from an overnight culture using the QIAGEN Genomic-tip 100/G kit and Genomic DNA Buffer Set (QIAGEN, Valencia, CA, USA) according to the manufacturer’s instructions. A Qubit dsDNA HS Assay kit and Qubit 2.0 fluorometer (Life Technologies, Carlsbad, CA, USA) were used to measure DNA concentration. A total of 2 µg of each DNA sample was used to build indexed PCR-free libraries using a multiplexed, high-throughput sequencing TruSeq DNA Sample Preparation Kit (Illumina, San Diego, CA, USA) according to the manufacturer’s protocols with minor modifications.

The genomic DNA of VGH116 and VGH117 were sequenced by Illumina MiSeq sequencer using paired-end (2 × 150 bp) sequencing, which generated 1,676,992 and 2,295,502 (69x) reads, respectively. The total read depth provided 51-fold and 69-fold coverage. VGH117 was additionally sequenced using Nanopore GridIon with 175-fold coverage (842 Mbp). Illumina read data were filtered using duk (http://duk.sourceforge.net/, accessed on 14 November 2020). Low quality (Q-score < 10) reads were trimmed, and a read was retained if at least 50 bp was obtained by the FASTQX-toolkit (https://github.com/agordon/fastx_toolkit, accessed on 14 November 2020). Nanopore read data were trimmed for low-quality bases via Porechop. The VGH116 genome was assembled using Velvet v. 1.2.07 and ALLPATHS v. R46652, using a 31 bp k-mer size (https://github.com/agordon/fastx_toolkit, accessed on 14 November 2020). The VGH117 genome was reconstructed using a hybrid Illumina and Nanopore assembly pipeline called Unicyler.

### 3.2. Annotation of Protein-Coding Genes, Virulence Factors, and Antibiotic Resistance

The VGH116/VGH117 genomes were annotated using the National Center for Biotechnology Information (NCBI) Prokaryotic Genomes Automatic Annotation Pipeline (PGAAP). Functional classification of the predicted genes was performed using the RPSBLAST program v. 2.2.15 in conjunction with the COGs (Clusters of Orthologous Groups of proteins) databases, using an E-value threshold <0.001. Virulence genes in the VGH116/VGH117 genomes were separately identified by running BLAST against the Virulence Factors Database (VFDB). The assembled genome was first aligned against the VFDB protein sequences of the full dataset (set B) under the following criteria: identity > 45%, aligned length > 450 bp, alignment coverage > 95%, and E-value < 1e−45. If multiple virulence factor genes overlapped at the same locus in the genome, only the best-aligned virulence factor gene was retained. Antibiotic-resistant genes were predicted via BLAST search against the Comprehensive Antibiotic Resistant Database (CARD) using the same criteria. The efflux pump genes were excluded from the analysis.

### 3.3. Phylogeny Reconstruction

We used OrthoANI to compute the Average Nucleotide Identity (ANI) among different species, as whole-genome ANI has now become the standard for species identification/confirmation by NCBI during genome submission. The OrthoANI reconstructs the phylogeny in different ways. First, the genome is chopped into pieces of 1020 bp and BLAST is used to compute the ANI of all homologous pairs. Finally, the unweighted pair group method with arithmetic mean (UPGMA) is invoked to cluster the species using the ANI as the distance metric.

## 4. Results

### Genome Features of VGH116 and VGH117

**Figure 1:**
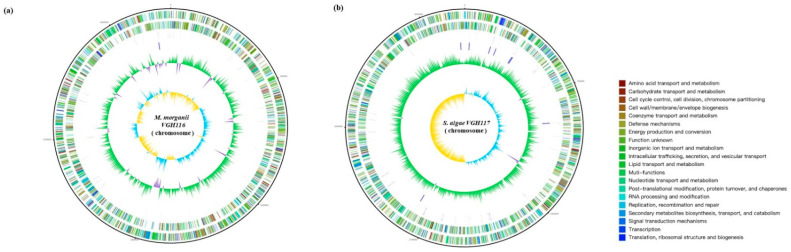
Circular genome maps of (**a**) VGH116 and (**b**) VGH117 genomes. Circles from the outside to inside showing: 1. DNA coordinates; 2,3. function-based color-coded mapping of the CDSs predicted on the forward and reverse strands. Functions are color-coded according to COG classifications; 4. tRNA genes; 5. rRNA genes; 6. GC plot showing regions above the average (green) and below (violet); 7. GC skew showing regions above the average (yellow) and below (light blue).

**Figure 2:**
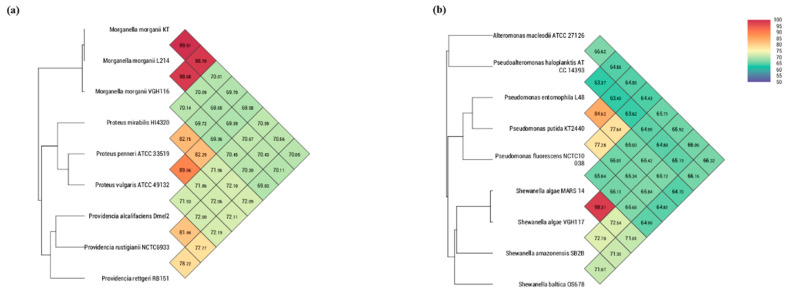
Genome-wide average nucleotide identities of (**a**) VGH116 and (**b**) VGH117 genomes against closely related species.

Whole-genome sequencing was performed over *M. morganii* VGH116 and *S. algae* VGH117 using the Illumina MiSeq and Oxford Nanopore GridIon at 58- to 175-fold coverage. (see Methods). Then de novo assembly was used to reconstruct a 3.8 Mbp genome for VGH116 and a 4.7 Mbp genome for VGH117 (Figure 1). The GC content was 50.89% for VGH116 and 53.44% for VGH117. Genome analysis indicated that *M. morganii* VGH116 was highly similar to the *M. morganii* L214 (>98% ANI), and S. *algae* VGH117 was close to *S. algae* MARS (>98% ANI) (Figure 2), confirming the species identification using 16S rRNA. We observed a strong GC skew of the VGH117 genome in which half of the chromosome was dominated by G+C, while the other half was enriched with A+C (Figure 1).

In total, 4371 and 4217 protein-coding genes and RNAs (including 64 rRNAs, tRNAs, and ncRNAs) were annotated in the VGH116 and VGH117 genomes (Table 2). Phenotypically, *M. morganii* VGH116 was resistant to first-generation cephalosporins (cefazolin MIC ≥64 mg/L) but was susceptible to ampicillin/sulbactam (MIC ≤2 mg/L), piperacillin/tazobactam (MIC ≤2 mg/L), third and fourth generation cephalosporins (ceftazidime, ceftriaxone, cefepime), imipenem, amikacin, gentamicin, ciprofloxacin, and trimethroprim–sulfamethoxazole. Analysis of the resistome of *M. morganii* VGH116 revealed the presence of antibiotic resistance genes (ARGs). Several beta-lactamases were found and confirmed by multiple databases (i.e., UniPro, CARD, Genbank, and NCBI nr), including the NmcA beta-lactamase, cysB, and the class C beta-lactamases, AmpC (DHA-1) and AmpH.

Moreover, S. *algae* VGH117 was susceptible to ampicillin–sulbactam, piperacillin–tazobactam, third and fourth generation cephalosporins (ceftazidime, ceftriaxone, cefepime), imipenem, amikacin, gentamicin, ciprofloxacin, and trimethoprim–sulfamethoxazole. The resistome of this S. *algae* VGH 117 included the Ambler class D β-lactamases-encoding *BlaOXA-55* gene.

## 5. Discussion

Envenomation resulting from snakebite frequently leads to extensive tissue destruction or an infected wound [7]. Infection may arise from inoculation of bacteria in the snake’s mouth at the time of the bite. The oral flora of snakes vary among different species and geographic locations, which may be influenced by venom properties and the fecal flora of the prey in different geographic regions [5,8,9]. Live prey are thought to defecate in the snake’s mouth while being ingested [10]. The mouth of *N. atra* harbors larger numbers of bacterial species associated with snakebite wound infections than crotaline or colubrid snake species [5,9].

Shek et al. reported that *M. morganii*, *Enterococcus faecalis*, and *Clostridium* species were the most commonly isolated pathogens in the oropharynx of *N. atra*. Only two of 32 oral flora of *N. atra* were identified as *S. putrefaciens*. In Taiwan [11,12], among the aerobic bacteria isolated from pathogenic organisms present at the site of a *N. atra* bite wound, *M. morganii* and *Enterococcus faecalis* were the most common, followed by *Proteus spp. Bacteroides fragilis* was an anerobic bacteria that was isolated. Mao et al. reported that only three of 112 cases were identified as *S. putrefaciens* [13].

In a previous report which reviewed all known cases of *N. atra* envenomation at Taichung and Taipei Veterans General Hospitals, 64% of cases had polymicrobial infection (≥ 2 pathogens), from which more than two organisms were isolated [13]. However, only a few studies have reported on *Shewanella* infections. *Shewanella* is probably an underestimated and underreported cause of wound infections following snake bites [13]. In a retrospective study of *Shewanella* infection of snake bites during a twelve-year period, Po-Yu Liu et al. reported that *Shewanella* infection was associated with moderate to severe local envenomation [14]. Coinfection with *S. algae* and *M. morganii* of wounds from the *N. atra* bite is rare. Furthermore, we isolated *S*. *algae* from the debrided wound and blood. *Shewanella* infections are increasingly being reported and have mostly been attributed to *S. putrefaciens*. However, recent data suggest that many *S. putrefaciens* isolates should be classified as the genetically distinct species *S*. *algae* rather than *S. putrefaciens*. This misclassification is largely due to the fact that only *S. putrefaciens* has been included in the databases of conventional systems. [15].

Previous reports on *M. morganii* and *Shewanella putrefaciens* isolated from cobra bite wounds showed that these bacteria were susceptible to amikacin, ceftazidime, ciprofloxacin, and piperacillin/tazobactam [16]. In our case, two antibiotic-resistance genes in the *M. morganii* VGH116 genome were *AmpC* and *AmpH. AmpC* encodes a chromosomal class C beta-lactamase. *AmpH* bound strongly to penicillin G, cefoxitin, and cephalosporin C; was temperature sensitive; and disappeared from cells after overnight incubation in the stationary phase [17]. Antibiotic-resistance genes with *Bla*_OXA-55_ were found in the *S. algae* VGH117 genome. *Bla*_OXA-55_ has a narrow spectrum of hydrolysis on penicillins and narrow-spectrum cephalosporins and imipenem [18]. In this case report, we isolated *M. morganiii* harboring *ampC* and *ampH*, which conferred resistance to cephamycin and the first-generation cephalosporin. *S. algae* strains harbored the quinolone-resistance-associated genes (*qnrA, gyrA, gyrB, parC*, and *parE*) regardless its resistance to ciprofloxacin. *S. algae* VGH117 also contained *qnrA, gyrA, gyrB* but had no resistance to quinolones [19].

The *S. algae* VGH117 genome showed prominent GC-skews, which have been proven to be useful as an indicator of the DNA leading strand, lagging strand, replication origin (ori), and replication terminal (ter) [20]. The two regions of GC-skew divide the genome into two halves, and the two breakpoints contain the replication origin (ori) and terminus (ter) sequences.

The oral cavity of *N. atra* harbors a wide range of pathogenic bacteria; however, bacterial coinfection with snakebites has not been common in previous studies. Snake venom toxins contributed to the development of wound infection, and the pathogenicity of microorganisms could further worsening the wound condition [21]. The bacterial composition may be altered before bacterial culture collection in a prehospital setting, during transportation, or during hospitalization. Additionally, many bacteria do not grow on the culture media commonly used, which led to the insufficient numbers of colonies. These limitations may have resulted in the uncommon case reports of the coinfection of *M. morganii* and *S. algae* following snakebites. Further studies are required to clarify such an issue.

## 6. Conclusions

In this study, we demonstrated *N. atra* bite wound infection caused by *M. morganii* carrying *AmpC* and *AmpH* and *S. algae* carrying *Bla*_OXA-55_. Empiric antibiotics for snake bite wound infection need to consider the reported pathogen of *N. atra* wound infection and associated resistance genes. With the rising of antimicrobial resistance [22], the application of whole genome sequencing in resistance determinants analysis will be a common practice.

## Figures and Tables

**Table 1:** Antimicrobial susceptibility testing of *M. morganii* VGH116 and *S. algae* VGH117.

	*M. morganii* VGH 116	*S. algae* VGH 117
	MIC (mg/L); Interpretation	
Cefazolin	64; R	NA
Ceftazidime	≤ 0.12; S	≤ 0.12; S
Ceftriaxone	≤ 0.25; S	≤ 0.25; S
Cefepime	≤ 0.12; S	≤ 0.12; S
Piperacillin–tazobactam	≤ 0.25; S	≤ 4; S
Imipenem	1; S	2; S
Gentamicin	≤ 1; S	≤ 1; S
Trimethoprim–sulfamethoxazole	≤ 20; S	≤ 20; S

MIC: minimum inhibitory concentration, S: susceptible; R: resistant; NA: not available.

**Table 2:** Genome and gene annotation statistics of VGH116 and VGH117 genomes.

Organism	Strain	Genome Size	GC Content	Genes (Coding)	Genes (RNA)
*Morganella morganii*	VGH116	3.8 Mbp	50.89%	4141	64
*Shewanella algae*	VGH117	4.7 Mbp	53.44%	4102	64

## Data Availability

Data relating to this study are presented within the manuscript. Other materials are available from the corresponding author upon reasonable request.
